# Aetiological phenotypes of atrial and ventricular secondary tricuspid regurgitation and their prognostic implications: insights from the CARE‐TR registry

**DOI:** 10.1002/ejhf.3678

**Published:** 2025-05-07

**Authors:** Laura Lupi, Elena Antonioli, Angelica Praderio, Alessandro Villaschi, Elisa Soranzo, Nicola Saccani, Kamil Stankowski, Daniele Cocianni, Beniamino Pagliaro, Valeria Magni, Marco Loffi, Daniela Tomasoni, Matteo Pagnesi, Antonio Mangieri, Davide Stolfo, Gianfranco Sinagra, Marianna Adamo, Marco Metra

**Affiliations:** ^1^ Institute of Cardiology, ASST Spedali Civili di Brescia, Department of Medical and Surgical Specialties, Radiological Sciences, and Public Health University of Brescia Brescia Italy; ^2^ Division of Cardiology ASST del Garda, Presidio Ospedaliero di Manerbio Manerbio Italy; ^3^ Department of Biomedical Sciences Humanitas University, Pieve Emanuele‐Milan, Italy; IRCCS Humanitas Research Hospital Rozzano‐Milan Italy; ^4^ Cardiothoracovascular Department Azienda Sanitaria Universitaria Giuliano Isontina (ASUGI) and University Hospital of Trieste Trieste Italy; ^5^ Division of Cardiology ASST del Garda, Presidio Ospedaliero di Desenzano del Garda Desenzano del Garda Italy; ^6^ Division of Cardiology Azienda Socio‐Sanitaria Territoriale di Cremona Cremona Italy; ^7^ Division of Cardiology, Department of Medicine Karolinska Institutet Stockholm Sweden

**Keywords:** Atrial secondary tricuspid regurgitation, Ventricular secondary tricuspid regurgitation, Aetiological phenotypes, Prevalence, Outcomes

## Abstract

**Aims:**

To report prevalence and clinical outcomes of different aetiological phenotypes of atrial and ventricular secondary tricuspid regurgitation (ASTR/VSTR).

**Methods and results:**

The Consecutive pAtients with seveRE Tricuspid Regurgitation evaluated in Heart Failure (HF) and Valve Clinics (CARE‐TR) registry collected data from patients with at least severe tricuspid regurgitation (TR) enrolled at three Italian centres. The present analysis includes 648 patients with secondary TR, 22.1% with ASTR and 77.9% with VSTR. Patients with ASTR were further stratified in those with atrial fibrillation (AF, 25.2%), HF with preserved ejection fraction (HFpEF, 37.8%), and both (37.0%). Patients with VSTR were subdivided into those with severe left‐sided valvular heart disease (LS‐VHD, 28.5%), HF with reduced or mildly reduced ejection fraction (HFrEF/HFmrEF, 29.1%), HFpEF (35.5%), pre‐capillary pulmonary hypertension (PH, 4.0%) and isolated right ventricular dysfunction (RVD, 2.9%). After a median follow‐up of 498 days, 118 (18.2%) patients died and 153 (23.6%) were hospitalized for HF. Two‐year survival free from the composite outcome of death or HF hospitalization was higher in patients with ASTR compared with those with VSTR (73.5% vs. 54.4%; *p* < 0.001). After adjustment for variables related with HF severity, VSTR remained independently associated with an increased risk of events (adjusted hazard ratio 2.00; 95% confidence interval 1.33–3.02; *p* = 0.001). Among ASTR patients, combined AF and HFpEF was associated with a poorer outcome compared with AF or HFpEF alone (60.2% vs. 80.5% vs. 83.6%; *p* = 0.022). Among patients with VSTR, overall survival free from the composite outcome was 85%, 65%, 54%, 39% and 38% for RVD, HFpEF, HFrEF/HFmrEF, severe LS‐VHD, and pre‐capillary PH, respectively (*p* < 0.001).

**Conclusions:**

In a real‐world population with at least severe secondary TR, 22% had ASTR and showed better outcomes as compared to VSTR. Among patients with ASTR, combination of AF and HFpEF was common and associated with the worst prognosis. Among patients with VSTR, those with pre‐capillary PH had the poorest outcomes, followed by those with LS‐VHD.

## Introduction

Tricuspid regurgitation (TR) is frequent in the general population and its prevalence is high in patients with heart failure (HF).^1^, ^2^, ^3^, ^4^ It is known to be associated with an increased risk of mortality and cardiovascular events regardless of disease stage and HF severity.^2^, ^3^, ^5^, ^6^, ^7^

New classifications based on its causes identify primary, cardiac implantable electronic device (CIED)‐related and secondary TR (STR).^8^, ^9^ STR represents more than 90% of clinically relevant TR and has two major subtypes: atrial and ventricular STR (ASTR/VSTR). VSTR is associated with right ventricular (RV) remodelling, including chamber dilatation and/or dysfunction, leading to predominant leaflet tethering and a variable extent of tricuspid annular dilatation. ASTR is characterized by normal leaflets that fail to coapt because of annular and atrial dilatation without significant tethering; RV structure is normal or mildly impaired due to basal enlargement and RV function is preserved. Discriminating ASTR from VSTR has strong prognostic implications since patient with VSTR have higher mortality than patients with ASTR.^6^, ^10^, ^11^, ^12^, ^13^ However, both ASTR and VSTR may have different causes and few data are available regarding the clinical characteristics and outcomes of different aetiological phenotypes of STR.^9^, ^10^, ^14^

The aim of this study is to better define aetiological phenotypes of ASTR and VSTR reporting their prevalence, clinical characteristics, and outcomes. Recognition of aetiological phenotypes associated with worse outcomes may be useful to identify patients who may benefit the most from treatment of STR.

## Methods

### Study population

The Consecutive pAtients with seveRE Tricuspid Regurgitation evaluated in Heart Failure and Valve Clinics (CARE‐TR) registry was a prospective observational registry collecting data from consecutive patients with at least severe TR evaluated at three Italian Heart Failure centres (ASST Spedali Civili di Brescia, Brescia; Ospedali Riuniti di Trieste, Trieste; IRCCS Istituto Clinico Humanitas, Rozzano‐Milan).

All consecutive patients undergoing transthoracic echocardiography between January 2020 and December 2022 with severe, massive or torrential TR were included, whereas those with active endocarditis and known congenital heart disease were excluded. For the purpose of the present analysis, only patients with STR were included, while those with primary TR and CIED‐related TR were excluded.

This study complied with the principles outlined in the Declaration of Helsinki and was approved by the Institutional review board of each centre.

### Data collection

Baseline demographic, clinical, laboratory and echocardiographic variables as well as medications were collected at the time of the diagnosis of severe STR. All echocardiographic images were recorded on digital media storage at each institution and were prospectively re‐analysed by local expert echocardiographers, blinded to patient's clinical data and outcome. Cardiac chamber quantification and evaluation of systolic and diastolic function were performed according to the most recent international guidelines of the American Society of Echocardiography (ASE) and the European Association of Cardiovascular Imaging (EACVI).^15^, ^16^, ^17^ All RV measurements were performed on a RV‐focused four‐chamber apical view. RV end‐diastolic mid diameter (RV EDD) and indexed end‐diastolic areas (RV EDAi) were used to identify RV dilatation. RV fractional area change (RV FAC) was derived from RV end‐systolic area and RV EDA. Additionally, RV myocardial deformation analysis was performed using Autostrain RV (TOMTEC‐ARENA 2020, TomTec Imaging System GmbH, Unterschleissheim, Germany); the RV free wall longitudinal strain (RVFWLS) was calculated by averaging the strain values of the three free wall segments, as recommended by the joint EACVI/ASE consensus document.^18^ Right atrial (RA) size was measured at end‐systole on an apical four‐chamber view, RA volume was calculated using the single‐plane disk summation method and indexed for body surface area. Moreover, integrative assessment of TR severity was performed through a multi‐parametric approach including qualitative, semi‐quantitative, and quantitative parameters and were graded into severe, massive, and torrential according to recent classification (Tricuspid Valve Academic Research Consortium).^14^

Pulmonary artery systolic pressure (PASP) was estimated from the TR jet peak velocity applying the Bernoulli equation and adding RA pressure. RA pressure was estimated based on the inferior vena cava diameter and its collapsibility during breathing.

### Definitions and outcomes

Patients with STR were stratified into two groups: ASTR and VSTR. ASTR was defined by the presence of all the following parameters: (i) left ventricular ejection fraction (LVEF) ≥50%^6^, ^11^, ^19^; (ii) PASP ≤50 mmHg^6^, ^13^; (iii) RV mid EDD ≤38 mm and/or RV EDAi <13 cm^2^/m^2^ ^11^, ^19^; (iv) RV FAC ≥35% and/or RVFWLS ≤ −17%.^19^, ^20^ The lack of at least one of these parameters identified the VSTR aetiology.

Patients with ASTR were further stratified into three groups: patients with history of atrial fibrillation (AF), patients affected by heart failure with preserved ejection fraction (HFpEF), and patients with both AF and HFpEF. Patients with VSTR were subdivided into the following groups: patients with severe left‐sided valvular heart disease (LS‐VHD), patients with heart failure with reduced (HFrEF) or mildly reduced ejection fraction (HFmrEF) and patients with HFpEF, both without severe LS‐VHD; patients with pre‐capillary pulmonary hypertension (PH) and patients with primary RV dysfunction (RVD), both without signs of left ventricular systolic or diastolic dysfunction. The Heart Failure Association (HFA)‐PEFF score was used to identify patients with HFpEF (i.e. HFA‐PEFF score ≥5).^21^

The study outcome was a composite of all‐cause death or HF hospitalization. Information concerning vital status and hospitalizations was obtained through review of electronic medical records, scheduled clinical evaluation or phone contact.

### Statistical analysis

The normal distribution of continuous variables was tested with Kolmogorov–Smirnov test. Continuous variables are reported as median (interquartile range [IQR]) and categorical variables are reported as counts and percentages. Difference between ASTR and VSTR were evaluated with the Mann–Whitney U test for continuous variables and the *χ* ^2^ or Fisher's exact tests for categorical variables, as appropriate. Difference among the three groups of ASTR and the five groups of VSTR were analysed with the Kruskal–Wallis test and the *χ* ^2^ test for continuous and categorical variables, respectively.

The independent prognostic value of VSTR versus ASTR and of different aetiological phenotypes of ASTR and VSTR was evaluated with a Cox proportional hazard regression analysis including in the multivariable model variables judged to be clinically relevant, such as age, sex, glomerular filtration rate (GFR), coronary artery disease, history of AF, prior hospitalization for HF, and loop diuretic use (history of AF was excluded from the analysis of ASTR subgroups since it identifies one of the three categories). The cumulative survival free from all‐cause mortality or HF hospitalization was estimated using the Kaplan–Meier method and compared by means of the log‐rank test. All statistical analyses were performed using the SPSS software, version 26 (SPSS Inc., Chicago, IL, USA). A two‐sided significance level of *p* < 0.05 was considered statistically significant.

## Results

### Prevalence of atrial and ventricular secondary tricuspid regurgitation

**Figure 1:**
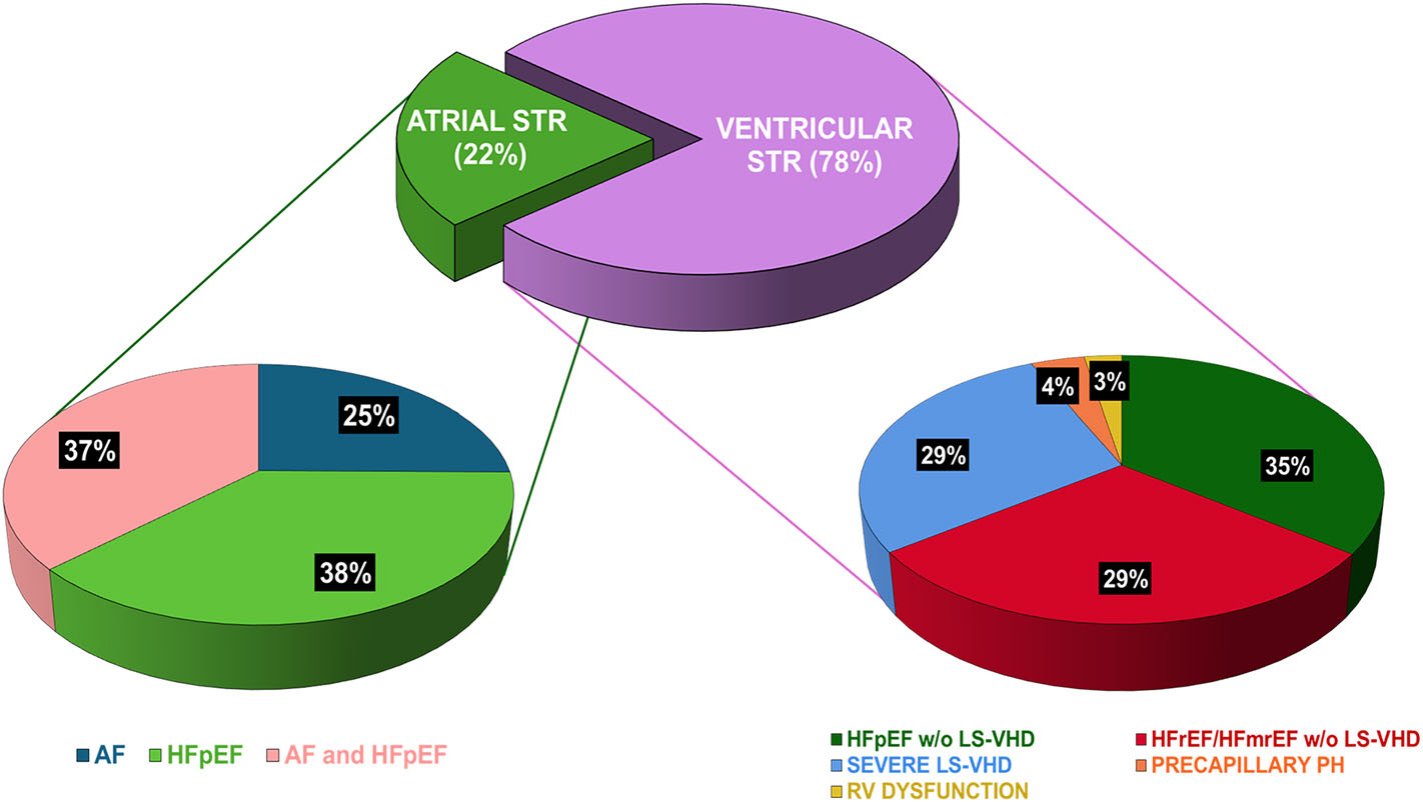
Distribution of secondary tricuspid regurgitation aetiological phenotypes. AF, atrial fibrillation; HFpEF, heart failure with preserved ejection fraction; HFmrEF, heart failure with mildly reduced ejection fraction; HFrEF, heart failure with reduced ejection fraction; LS‐VHD, left‐sided valvular heart disease; PH, pulmonary hypertension; RV, right ventricular; STR, secondary tricuspid regurgitation.

Among 737 patients included in the CARE‐TR registry, 25 had primary TR, 39 had CIED‐related TR and 25 were excluded due to missing data for at least one of the criteria to define ASTR. Thus, a total of 648 patients were included in the current analysis: 143 (22.1%) were classified as ASTR and 505 (77.9%) as VSTR. Among patients with ASTR, 36 (25.2%) had AF, 54 (37.8%) had HFpEF and 53 (37.0%) had both. Among VSTR, 144 (28.5%) had severe LS‐VHD, 147 (29.1%) had HFrEF or HFmrEF without severe LS‐VHD, 179 (35.5%) had HFpEF without severe LS‐VHD, 20 (4.0%) had pre‐capillary PH, and 14 (2.9%) had isolated RVD (*Figure* *1*).

### Baseline characteristics according to aetiological phenotype

Baseline demographic and clinical characteristics of the whole population and stratified by ASTR versus VSTR are summarized in *Table* *1*. Compared to patients with ASTR, those with VSTR were more likely to be male, to have cardiovascular comorbidities (i.e. coronary artery disease, history of stroke, peripheral artery disease) and a history of HF hospitalization. They also had worse kidney function, as estimated by GFR, and higher N‐terminal pro‐B‐type natriuretic peptide (NT‐proBNP) plasma concentrations, worse New York Heart Association (NYHA) functional class and received higher doses of loop diuretics. As well, they showed higher TRI‐SCORE values.

**Table 1:** Demographic, clinical and laboratory characteristics of the overall population and of patients with atrial versus ventricular secondary tricuspid regurgitation

	All patients (*n* = 648)	Atrial STR (*n* = 143)	Ventricular STR (*n* = 505)	*p*‐value
Age (years)	80 [72–84]	80 [72–84]	80 [72–84]	0.926
Male sex	292 (45.1)	40 (27.9)	252 (49.9)	**<0.001**
Body surface area (m^2^)	1.79 [1.62–1.92]	1.75 [1.59–1.87]	1.79 [1.62–1.94]	0.053
Hypertension	423 (65.2)	83 (58.0)	340 (67.3)	**0.026**
Diabetes	154 (23.8)	27 (18.8)	127 (25.1)	0.073
Dyslipidaemia	271 (41.8)	50 (34.9)	221 (43.8)	**0.036**
Cancer	43 (6.6)	7 (4.9)	36 (7.1)	0.229
COPD	104 (16.0)	14 (9.8)	90 (17.8)	**0.012**
CAD	161 (24.8)	22 (15.4)	139 (27.5)	**0.002**
Prior PCI	113 (17.4)	13 (9.1)	100 (19.8)	**0.001**
Prior CABG	69 (10.6)	11 (7.7)	58 (11.5)	0.124
Prior surgical valve intervention	13 (2.0)	1 (0.7)	12 (2.4)	0.317
Prior transcatheter valve intervention	11 (1.7)	5 (3.5)	6 (1.2)	0.071
History of stroke/TIA	78 (12.0)	10 (7.0)	68 (13.5)	**0.021**
PAD	146 (22.5)	21 (14.7)	125 (24.7)	**0.006**
History of atrial fibrillation	445 (68.7)	89 (62.2)	356 (70.5)	**0.012**
Type of atrial fibrillation				
Paroxysmal	116 (26.1)	28 (31.5)	88 (24.7)	0.224
Long‐standing	329 (73.9)	61 (68.5)	268 (75.3)	
Prior HF hospitalization	190 (29.3)^a^	32 (22.4)^a^	158 (31.3)^a^	**0.025**
CRT‐P	16 (2.5)	3 (2.1)	13 (2.6)	0.515
CRT‐D	41 (6.3)	3 (2.1)	38 (7.5)	**0.010**
PM	117 (18)	23 (16.1)	94 (18.6)	0.287
ICD	38 (5.9)	2 (1.4)	36 (7.2)	**0.004**
Creatinine (mg/dl)	1.2 [0.92–1.73]	1.1 [0.85–1.47]	1.3 [0.96–1.85]	**<0.001**
GFR CKD‐EPI (ml/min)	46.0 [31.0–69.2]	56.0 [38–74.4]	44.9 [29.3–66.5]	**0.001**
Haemoglobin (g/dl)	12.1 [10.6–13.2]	12.3 [11–13.4]	11.9 [10.4–13.2]	**0.040**
BNP (ng/L)	430 [235–852]	261.5 [151.5–406]	431 [238–862]	0.101
NT‐proBNP (ng/L)	2898 [1332–7111]	1525 [618–2746]	3483 [1663–8558]	**<0.001**
Total bilirubin (mg/dl)	0.83 [0.65–1.29]	0.82 [0.5–1.35]	0.83 [0.68–1.24]	0.554
NYHA class				
I	127 (19.6)	35 (24.5)	92 (18.2)	**<0.001**
II	323 (49.8)	86 (60.1)	237 (46.9)	
III	169 (26.1)	19 (13.3)	150 (29.7)	
IV	29 (4.5)	3 (2.1)	26 (5.2)	
Loop diuretics	579 (89.5)^a^	120 (84.5)^a^	459 (90.9)	**0.043**
Loop diuretic dosage (mg)	50.0 [25.0–100]	50.0 [25.0–75.0]	50.0 [25.0–100]	**<0.001**
Beta‐blockers	335 (83.3)^a^	75 (69.4)^a^	260 (88.4)^a^	**<0.001**
ACEi/ARB/ARNI	208 (52.0)^a^	41 (37.9)^a^	167 (57.2)^a^	**0.001**
MRA	186 (46.5)^a^	29 (26.7)^a^	157 (53.8)^a^	**<0.001**
SGLT2 inhibitors^b^	16 (4.0)^a^	0	16 (54.4)^a^	**0.008**
HFA‐PEFF score	6.0 [5.0–6.0]	5.5 [4.0–6.0]	6.0 [5.0–6.0]	**<0.001**
TRI‐score	3 [2–5]	2 [1–3]	4 [2–5]	**<0.001**

Values are expressed as *n* (%) or median [interquartile range], as appropriate.

ACEi, angiotensin‐converting enzyme inhibitor; ARB, angiotensin II receptor blocker; ARNI, angiotensin receptor–neprilysin inhibitor; BSA, body surface area; CABG, coronary artery bypass graft; CAD, coronary artery disease; CKD‐EPI, Chronic Kidney Disease Epidemiology Collaboration; COPD, chronic obstructive pulmonary disease; CRT‐D/P, cardiac resynchronization therapy with defibrillator/pacemaker; GFR, glomerular filtration rate; HF, heart failure; HFA‐PEFF score, Heart Failure Association‐PEFF score; ICD, implantable cardioverter‐defibrillator; MRA, mineralocorticoid receptor antagonist; NT‐proBNP, N‐terminal pro‐B‐type natriuretic peptide; NYHA, New York Heart Association; PAD, peripheral artery disease; PCI, percutaneous coronary intervention; PM, pacemaker; SGLT2, sodium–glucose cotransporter 2; STR, secondary tricuspid regurgitation; TIA, transient ischaemic attack.

^a^ The proportion of patients was calculated on the number of patients with available data.

^b^ Most patients were included in the registry prior to Italian Medicines Agency approval of SGLT2 inhibitors for HF.

The echocardiographic characteristics of the whole population and stratified by ASTR and VSTR are shown in *Table* *2*. As expected, patients with VSTR showed lower median values of LVEF, higher RV dimensions, worse RV function evaluated by FAC and RVFWLS, higher tricuspid tenting height, and higher PASP values, compared to patients with ASTR. They also had larger left ventricles, left atria and right atria. No significant differences were observed in tricuspid annular dimensions and in TR severity between the two groups.

**Table 2:** Echocardiographic characteristics of the overall population and of patients with atrial versus ventricular secondary tricuspid regurgitation

	All patients (*n* = 648)	Atrial STR (*n* = 143)	Ventricular STR (*n* = 505)	*p*‐value
LVEDD (mm)	50.0 [45.0–56.0]	48.0 [44.0–52.0]	52.0 [46.0–58.0]	**<0.001**
LVESD (mm)	36.0 [29.0–43.0]	31.0 [27.0–37.0]	38.0 [31.0–45.0]	**<0.001**
LVEDV index (ml/m^2^)	53.1 [42.0–74.0]	47.0 [40.0–58.5]	56.7 [43.0–77.0]	**<0.001**
LVESV index (ml/m^2^)	24.0 [17–38]	19.0 [15.0–25.0]	27.0 [19.0–45.0]	**<0.001**
LV ejection fraction (%)	54 [42–60]	60 [55–63]	50 [37–59]	**<0.001**
LVM index (g/m^2^)	123.0 [97.8–148.2]	112.0 [90.0–132.0]	128.0 [101.0–156.0]	**<0.001**
MR grade (%)				
None	32 (4.9)	5 (3.5)	27 (5.3)	0.480
Mild	160 (24.7)	29 (20.2)	131 (25.9)	
Moderate	189 (29.2)	43 (30.1)	146 (28.9)	
Moderate–severe	114 (17.6)	39 (27.3)	87 (17.2)	
Severe	153 (23.6)	27 (18.9)	114 (22.5)	
Mechanism of MR				
Primary	96 (60.1)^a^	40 (42.5)^a^	56 (17.9)^a^	**<0.001**
Secondary	247 (60.1)^a^	35 (37.2)^a^	212 (67.7)^a^	
Mixed	64 (15.7)^a^	19 (20.2)^a^	45 (14.4)^a^	
MR EROA‐PISA (cm^2^)	0.32 [0.22–0.41]	0.4 [0.26–0.51]	0.32 [0.22–0.4]	0.151
MR vena contracta (mm)	5.0 [3.0–6.3]	5.0 [3.0–6.8]	5.0 [3.0–6.3]	0.973
Moderate or severe aortic stenosis	43 (6.6)	13 (9.1)	30 (5.9)	0.117
Aortic regurgitation				
None	250 (39.2)^a^	52 (36.3)	198 (40.0)^a^	0.144
Mild	235 (36.8)^a^	46 (32.2)	189 (38.2)^a^	
Moderate	114 (17.9)^a^	32 (22.4)	82 (16.6)^a^	
Moderate–severe	24 (3.8)^a^	9 (6.3)	15 (3.0)^a^	
Severe	15 (2.3)^a^	4 (2.8)	11 (2.2)^a^	
Left atrial volume index (ml/m^2^)	59.3 [45.0–77.6]	54.0 [42.9–70.5]	60.0 [47.0–79.0]	**0.016**
E/e′ average	13.0 [10.0–18.0]	11.0 [9.0–15.0]	14.0 [10.0–19.0]	**<0.001**
TR grade				
Severe	546 (84.2)	128 (89.5)	418 (82.7)	0.116
Massive	98 (15.1)	15 (10.5)	83 (16.4)	
Torrential	4 (0.62)	0 (0)	4 (0.8)	
TR EROA‐PISA (cm^2^)	0.43 [0.35–0.53]	0.42 [0.37–0.56]	0.44 [0.35–0.52]	0.895
TR vena contracta (mm)	7.200 [6.0–9.0]	7.8 [6.5–9.0]	7.5 [6.5–9.0]	0.421
TR regurgitant volume (ml)	41.0 [35.0–51.7]	46.0 [36.0–50.0]	45.0 [35.0–52.0]	0.625
AP tricuspid annular diameter (mm)	40.0 [38.0–44.0]	41.0 [39.0–43.0]	40.0 [38.0–44.0]	0.662
AP indexed tricuspid annular diameter (mm/m^2^)	23.0 [20.2–25.3]	23.1 [20.7–25.3]	23.0 [20.3–25.3]	0.671
SL tricuspid annular diameter (mm)	40.0 [38.0–44.0]	40.0 [39.0–43.0]	40.0 [38.0–44.0]	0.826
SL indexed tricuspid annular diameter (mm/m^2^)	22.6 [20.5–25.6]	23.5 [21.2–25.1]	22.8 [20.6–25.4]	0.365
Tricuspid tenting height (mm)	7.0 [5.0–9.0]	2.0 [1.0–4.0]	8.0 [6.0–10.0]	**<0.001**
Right atrial volume index (ml/m^2^)	48.0 [39.0–65.0]	45.0 [40.0–56.1]	51.0 [38.0–69.0]	**<0.001**
RV diastolic basal diameter (mm)	41.0 [37.0–46.0]	38.0 [35.0–40.0]	42.0 [38.0–47.0]	**<0.001**
RV diastolic mid diameter (mm)	34.0 [29.0–38.0]	32.0 [28.0–34.0]	35.0 [30.0–40.0]	**<0.001**
End‐diastolic area index (cm^2^/m^2^)	11.0 [9.0–13.0]	9.5 [8.0–10.4]	12.0 [10.0–14.0]	**<0.001**
TAPSE (mm)	18.0 [16.0–21.0]	20.0 [18.0–23.0]	17.0 [15.0–20.0]	**<0.001**
S' TDI (cm/s)	10.0 [9.0–12.0]	12.0 [10.0–13.0]	10.0 [8.0–12.0]	**<0.001**
RVFWLS (%)	−18.0 [−23 to −13]	−24.0 [−27 to −20]	−15.0 [−20 to −12]	**<0.001**
Fractional area change (%)	41.0 [36.0–45.0]	45.0 [41.0–49.0]	40.0 [35.0–44.0]	**<0.001**
PASP (mmHg)	45.0 [38.0–55.0]	40.0 [35.0–45.0]	50.0 [40.0–60.0]	**<0.001**
RAP (mmHg)	10.0 [5.0–15.0]	5.0 [5.0–10.0]	10.0 [5.0–15.0]	**<0.001**

Values are expressed as *n* (%) or median [interquartile range], as appropriate.

AP, antero‐posterior; EROA‐PISA, effective regurgitant orifice area‐proximal isovelocity surface area; LV, left ventricle; LVEDD, left ventricular end‐diastolic diameter; LVEDV, left ventricular end‐diastolic volume; LVESD, left ventricular end‐systolic diameter; LVESD, left ventricular end‐systolic diamete; LVM, left ventricular mass; MR, mitral regurgitation; RAP, right atrial pressure; RV, right ventricle; RVFWLS, right ventricular free wall longitudinal strain; S′ TDI, Doppler tissue imaging‐derived tricuspid lateral annular systolic velocity; SL, septal‐lateral; PASP, pulmonary artery systolic pressure; TAPSE, tricuspid annular plane systolic excursion; TR, tricuspid regurgitation.

^a^ The proportion of patients was calculated on the number of patients with available data.

Baseline characteristics of the ASTR and VSTR further stratified by different aetiological phenotypes are reported in online supplementary *Tables* *S1* and *S2*. Patients with both AF and HFpEF were more likely to have history of hypertension, had higher values of NT‐proBNP and NYHA functional class III and IV compared to those with either AF or HFpEF alone. Furthermore, they had more enlarged atria, higher E/e′ average values, worse RV function, higher estimated PASP and were more likely to have massive TR, as compared to patients with isolated AF or HFpEF.

Among patients with VSTR, those with pre‐capillary PH were more frequently female and were more likely to have history of chronic obstructive pulmonary disease and higher values of NT‐proBNP as compared to the other VSTR subgroups. Also, they had smaller left‐sided chambers, worse RV dimension and function, higher indexed annular dimension, tenting height, and PASP values. Moreover, patients with severe LS‐VHD, pre‐capillary PH and HFrEF/HFmrEF showed higher TRI‐SCORE values than the other aetiological phenotypes. No significant differences in TR severity were noted between the five groups.

### Outcomes according to aetiological phenotype

**Figure 2:**
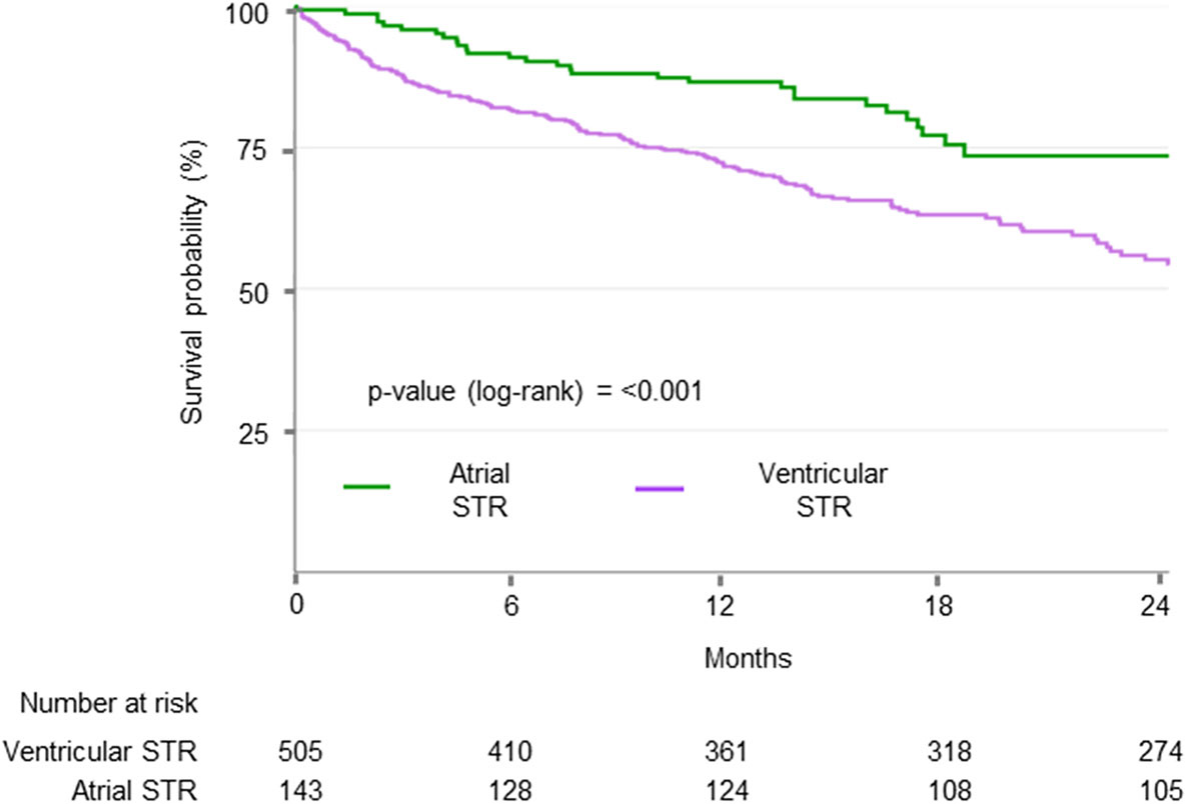
Kaplan–Meier curves for cumulative survival free from the composite outcome according to atrial and ventricular secondary tricuspid regurgitation (STR).

After a median follow‐up of 498 days (IQR 379–676), 118 (18.2%) patients died and 153 (23.6%) were hospitalized for HF. Two‐year survival free from the composite outcome was higher in patients with ASTR, as compared to those with VSTR (73.5% vs. 54.4%; log‐rank *p* < 0.001) (*Figure* *2*). VSTR was associated with an increased risk of the composite endpoint regardless of demographic and clinical variables (adjusted hazard ratio [HR] 2.00; 95% confidence interval [CI] 1.33–3.02; *p* = 0.001).

**Figure 3:**
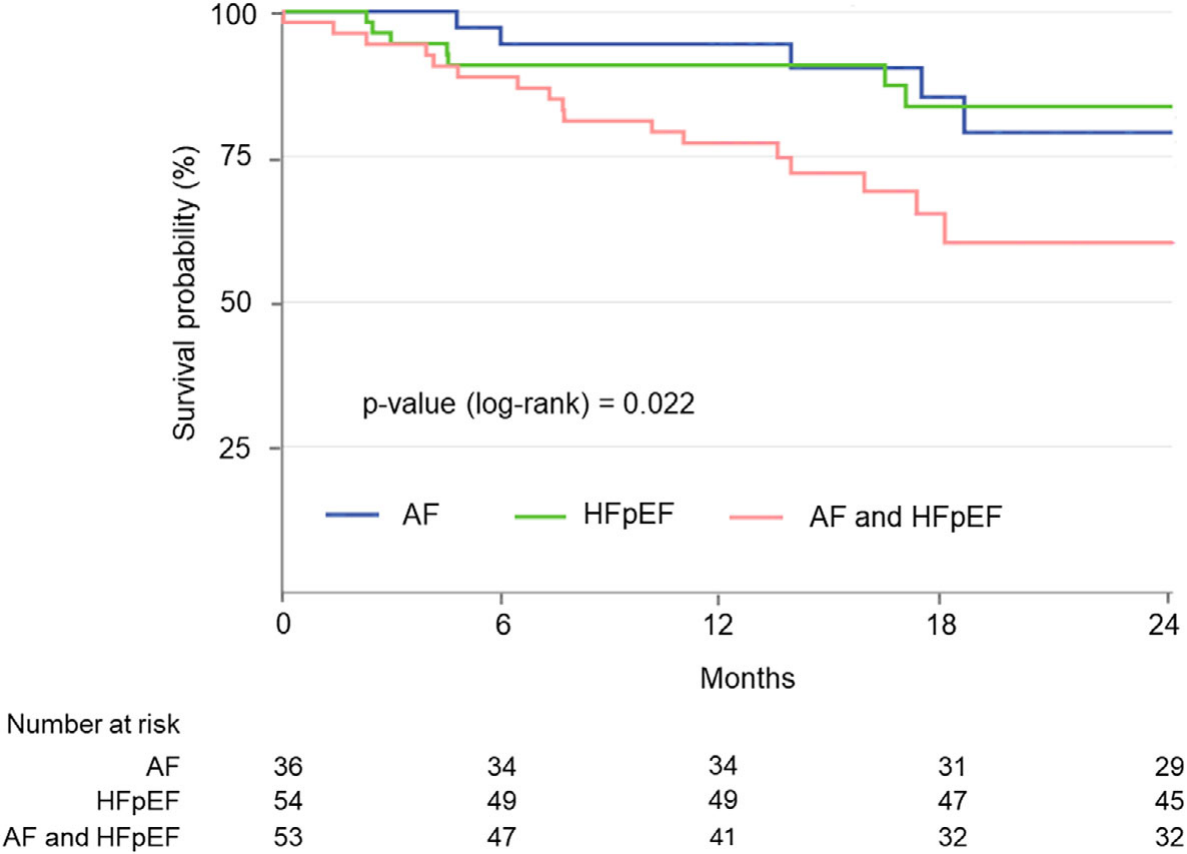
Kaplan–Meier curves for cumulative survival free from the composite outcome according to atrial secondary tricuspid regurgitation aetiological phenotypes. AF, atrial fibrillation, HFpEF, heart failure with preserved ejection fraction.

Among patients with ASTR, 2‐year survival free from the composite outcome was lower in patients with both AF and HFpEF, compared with those having either AF or HFpEF (60.2% vs. 80.5% vs. 83.6%, log‐rank *p* = 0.022) (*Figure* *3*). Patients with combined AF and HFpEF showed an independently higher risk of all‐cause death or hospitalization for HF compared with patients with isolated AF or HFpEF (adjusted HR 2.36; 95% CI 1.09–5.14, *p* = 0.030).

**Figure 4:**
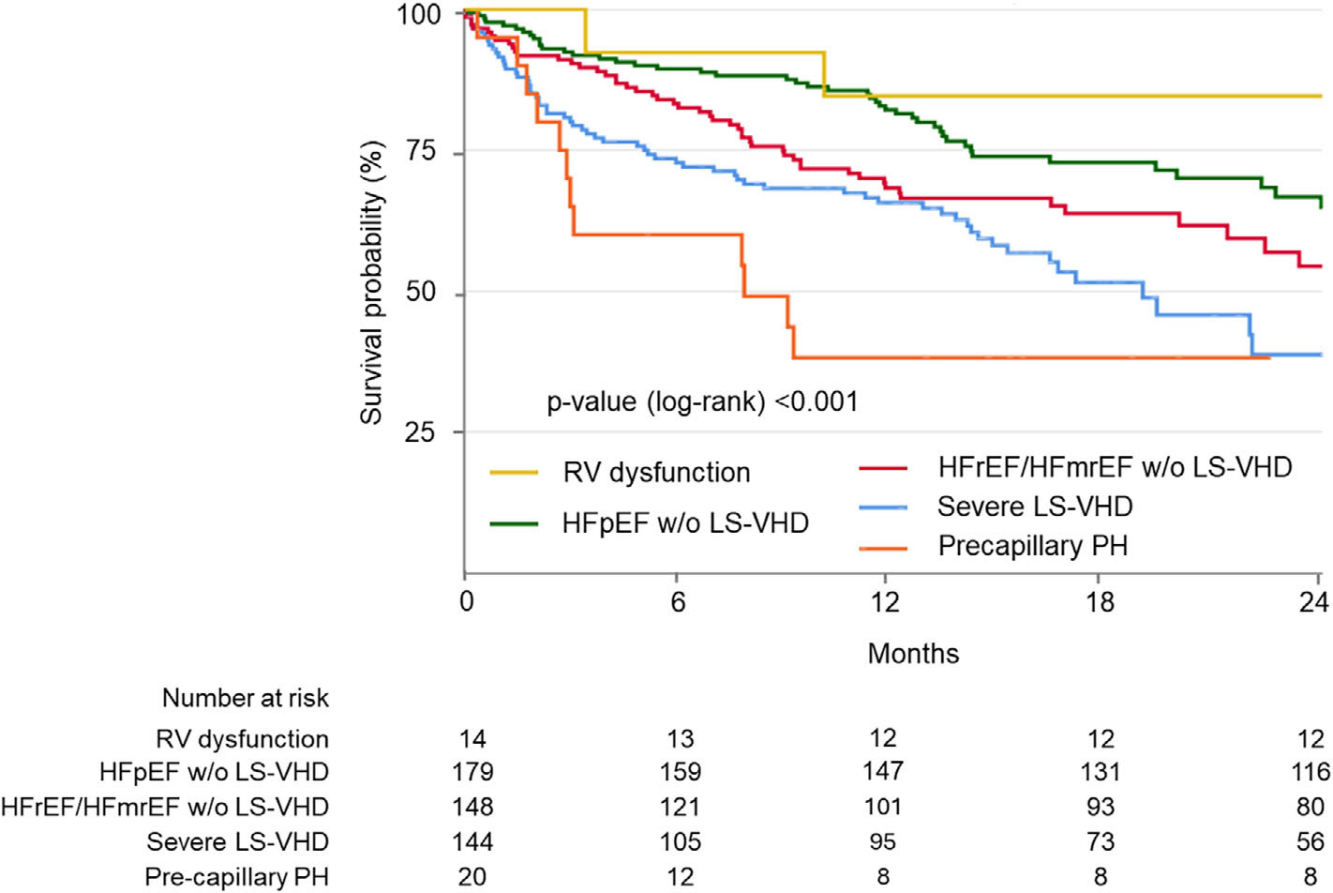
Kaplan–Meier curves for cumulative survival free from the composite outcome according to ventricular secondary tricuspid regurgitation aetiological phenotypes. HFmrEF, heart failure with mildly reduced ejection fraction; HFpEF, heart failure with preserved ejection fraction; HFrEF, heart failure with reduced ejection fraction; LS‐VHD, left‐sided valvular heart disease; PH, pulmonary hypertension; RV, right ventricular.

Among patients with VSTR, 2‐year survival free from the composite outcome was 85% in patients with RVD, 65% in patients with HFpEF without severe LS‐VHD, 54% in patients with HFrEF and HFmrEF without severe LS‐VHD, 39% in those with severe LS‐VHD, and 38% in patients with pre‐capillary PH (log‐rank *p* < 0.001) (*Figure* *4*). Pre‐capillary PH was associated with an independently increased risk of the composite endpoint compared with the other subgroups of VSTR (adjusted HR 2.85; 95% CI 1.56–5.18, *p* = 0.001).

Tricuspid valve procedures were performed in 67 patients during the follow‐up period: 50 patients underwent surgical intervention on the mitral valve (mitral valve repair in 47 patients and mitral valve replacement in 3 patients) and simultaneous tricuspid annuloplasty, 6 patients experienced mitral transcatheter edge‐to‐edge repair (TEER) and subsequent tricuspid TEER, 10 patients received isolated TEER, and one patient underwent surgical mitral and tricuspid valve replacement. Patient outcomes according to different aetiological phenotypes of STR were superimposable even including in the analysis only the 581 patients managed conservatively (online supplementary *Figures* *S1–S3*).

## Discussion

The main findings of the present study can be summarized as follows: (i) in a large, contemporary, multicentre, real‐world population with severe or higher grade secondary TR, prevalence of ASTR was 22%; (ii) patients with ASTR had a better prognosis, as compared to those with VSTR; (iii) among ASTR patients, combination of AF and HFpEF was common and associated with the worst outcome; and (iv) among VSTR patients, pre‐capillary PH was rare but associated with the poorest outcome, followed by LS‐VHD (Graphical Abstract).

Recent European and American expert position papers proposed a new integrated classification of TR with ASTR reported as a specific subgroup of STR and defined by the presence of RA enlargement and dysfunction leading to significant isolated annular dilatation with almost normal RV size and function.^1^, ^8^, ^14^, ^19^ However, a high heterogeneity in the specific definition of ASTR can be noted in previous studies. Schlotter *et al*.^11^ reported a prevalence of ASTR of 13.2% in a patient population with severe TR receiving either medical therapy or tricuspid TEER. They used the following definition of ASTR, based on a cluster analysis: tenting height ≤10 mm, RV mid ventricular diameter ≤38 mm and LVEF ≥50%. Galloo *et al*.^12^ showed a prevalence of 23.3% in patients with TR and identified ASTR by exclusion, namely as absence of RVD, PH and LS‐VHD. Importantly, only patients in sinus rhythm were considered as VSTR. Gavazzoni *et al*.^13^ defined ASTR as LVEF >60%, AF, PASP <50 mmHg, absence of left‐sided heart disease and normal tricuspid leaflets. They reported a prevalence of 26% in patients with both moderate and severe TR treated with medical therapy. Finally, Russo *et al*.^6^ recently reported a prevalence of ASTR, defined as LVEF >50%, AF, and PASP <50 mmHg, of 21.8% in patients receiving transcatheter tricuspid valve interventions.

Our definition of TR includes most of the criteria previously reported, namely absence of PH and left ventricular dysfunction as well as absence of moderate–severe RV dilatation and dysfunction in line with the recently proposed definitions.^14^, ^20^ Accordingly, we found a prevalence of ASTR of 22.1%. Regarding the clinical characteristics of patients with ASTR, previous studies consistently reported a high prevalence of female sex, less symptoms, and less comorbidities of ASTR versus VSTR.^11^, ^12^, ^13^ Our results were consistent with these findings. Small dissimilarities in the echocardiographic findings can be explained by the different criteria used to define ASTR. In particular, compared to other studies including RV dilatation and dysfunction in the definition of TR aetiology,^11^, ^12^ we included more accurate echocardiographic parameters, such as RV EDA and RVFWLS.

Only in two studies the presence of moderate–severe or severe mitral regurgitation (MR) was an exclusion criterion for the definition of ASTR.^12^, ^13^ However, when significant MR and TR are combined in the absence of high left ventricular filling pressure, PH, RV dilatation and/or dysfunction, and in the presence of RA and annular remodelling, STR could be considered as ASTR.

Previous studies consistently reported a better outcome of ASTR versus VSTR.^6^, ^11^, ^12^, ^13^ We confirmed these previous findings showing a two‐fold increased risk of all‐cause death or HF hospitalization in patients with VSTR versus those with ASTR.

A novel aspect of our study is the stratification of both ASTR and VSTR in subgroups based on their aetiologies. We proposed three aetiological phenotypes of ASTR based on the presence of AF and/or HFpEF. Permanent AF is known to be associated with increased risk of developing TR.^22^ The association between ASTR and AF is still debated since AF was considered as a criterion for the definition of ASTR in several previous studies,^6^, ^13^ but not in others.^11^, ^12^ As an example, in the study by Schlotter *et al*.^11^ 61% of patients with ASTR did not have AF. Furthermore, in recent expert opinion papers, AF was not considered mandatory in the definition of ASTR.^8^, ^14^, ^19^ Also, TR is known to be a common comorbidity in HFpEF patients, even in the absence of AF.^23^ A new concept of atrial myopathy has recently been recognized and may explain a portion of patients with no known history of AF, no clear HFpEF diagnostic criteria but isolated ASTR.^19^ In our study we did not find any patient who fit this last category. Maybe an even larger population is needed to clarify this evidence gap. The current study showed that both AF and HFpEF were common among patients with severe or greater ASTR (37%) and their coexistence identified the patients who had the worst prognosis. This is a novel finding of potential value when designing trials aimed at showing a beneficial effect on outcomes of TR treatment.

We also identified five phenotypes of VSTR based on specific aetiologies. Galloo *et al*.^12^ also stratified VSTR into left‐sided cardiac disease, PH and RVD and identified pre‐capillary PH as the worst and RVD as the best prognostic profile. We confirmed these results; however, in addition to Galloo *et al*.^12^ we further stratified left‐sided cardiac disease, which was the larger group, in HFpEF, HFrEF/HFmrEF and severe LS‐VHD. Among these patients, we identified severe LS‐VHD as the condition associated with the worst prognosis.

Our classification based on aetiologies represents a simple tool that may help in identifying STR patients at higher risk of unfavourable outcome. Further research implementing this stratification in different TR populations receiving different therapeutic options may guide a tailored patient selection for specific treatments (i.e. medical therapy vs. transcatheter interventions or surgery).

### Study limitations

Several limitations should be acknowledged. First, the relatively small sample size may have impacted on the robustness of our survival analyses. Second, the lack of a core laboratory may have limited our echocardiography‐based STR phenotyping and, consequently, may have impacted on the study findings. Third, data concerning left ventricular global longitudinal strain were not available for a large number of patients and were not included in the analysis. Fourth, data on right heart catheterization are not reported as available in only 15% of population. Fifth, some subgroups included a relatively small number of patients, thus limiting the power of the sub‐analyses. Finally, given the design of the study it is not possible to demonstrate a certain causative relationship between comorbidities (i.e. AF, HFpEF, HFrEF, HFmrEF, LS‐VHD, pre‐capillary PH or RVD) and STR; moreover, some patients may have been misclassified when having overlapping STR aetiologies or in advanced disease stages.

## Conclusions

In a large, contemporary, multicentre, real‐world population with at least severe STR, ASTR was observed in 22% of patients and was associated with better outcomes as compared to VSTR. Among ASTR patients, those with both AF and HFpEF were common and had the worst prognosis. Among VSTR patients, those with pre‐capillary PH had the worst prognosis, followed by patients with severe LS‐VHD.

## Supporting information


**Appendix S1.** Supporting Information.
