# Supplementary material for: Aetiological phenotypes of atrial and ventricular secondary tricuspid regurgitation and their prognostic implications: insights from the CARE‐TR registry
Source: Eur J Heart Fail. 2025 May 7;27(8):1549–58. doi: 10.1002/ejhf.3678 (PMC12482845; doi:10.1002/ejhf.3678)

**SUPPLEMENTAL MATERIALS**

**Aetiological phenotypes of atrial and ventricular secondary tricuspid regurgitation and their prognostic implications: insights from the CARE-TR registry**

**Supplemental Table 1. Demographic, clinical, laboratory and echocardiographic characteristics of the three aetiological phenotypes of atrial secondary tricuspid regurgitation.**

|  | **All ASTR patients**  **(n=143)** | **AF**  **(n=36)** | **HFpEF**  **(n=54)** | **AF and HFpEF**  **(n=53)** | **P value** |
| --- | --- | --- | --- | --- | --- |
| Age (years) | 80 [72-84] | 79.5 [72.2-86.5] | 77,5 [67.0-82.2] | 81.0 [75.5-84.0] | 0.074 |
| Male Sex | 40 (27.9) | 11 (30.5) | 14 (25.9) | 15 (28.3) | 0.889 |
| Body Surface Area (m^2^) | 1.75 [1.59-1.87] | 1.72 [1.63-1.89] | 1.71 [1.53-1.85] | 1.82 [1.63-1.95] | 0.082 |
| Hypertension | 83 (58) | 18 (50) | 26 (48.1) | 39 (73.6) | **0.015** |
| Diabetes | 27 (19) | 9 (25) | 10 (18.5) | 8 (15.1) | 0.501 |
| Dyslipidemia | 50 (35) | 11 (30.5) | 18 (33.3) | 21 (36.6) | 0.645 |
| Cancer | 7 (4.9) | 1 (2.8) | 3 (5.5) | 3 (5.6) | 0.793 |
| COPD | 14 (9.8) | 4 (11.1) | 3 (5.5) | 7 (13.2) | 0.393 |
| CAD | 22 (15.4) | 4 (11.1) | 13 (24.1) | 5 (9.4) | 0.079 |
| Prior PCI | 13 (9.1) | 2 (5.5) | 9 (16.6) | 2 (3.8) | **0.047** |
| Prior CABG | 11 (7.7) | 2 (5.5) | 6 (11.1) | 3 (5.6) | 0.490 |
| Prior surgical valve intervention | *1 (0.7)* | 0 | 1 (1.8) | 0 | 0.436 |
| Prior transcatheter valve intervention | *5 (3.5)* | 0 | 2 (3.7) | 3 (5.7) | 0.359 |
| History of Stroke/TIA | 10 (7.0) | 2 (5.5) | 2 (3.7) | 6 (11.3) | 0.281 |
| PAD | 21 (14.7) | 5 (13.9) | 11 (20.4) | 5 (9.4) | 0.276 |
| History of Atrial Fibrillation | 89 (62.2) | 36 (100) | 0 | 53 (100) | **<0.001** |
| Type of Atrial Fibrillation  - Paroxysmal  - Long standing | 28 (31.5)  61 (68.5) | 19 (52.7)  17 (47.3) | 0  0 | 9 (17.0)  44 (83.0) | **<0.001** |
| Prior HF Hospitalization | 32 (22.4) | 7 (19.4) | 14 (25.9) | 11 (20.8) | 0.722 |
| CRT-P | 3 (2.1) | 1 (2.8) | 1 (1.8) | 1 (1.9) | 0.947 |
| CRT-D | 3 (2.1) | 2 (5.5) | 1 (1.8) | 0 | 0.197 |
| PM | 23 (16.1) | 8 (22.2) | 3 (5.5) | 12 (22.6) | **0.028** |
| ICD | 2 (1.4) | 2 (5.5) | 0 | 0 | **0.049** |
| Admission creatinine (mg/dl) | 1.1 [0.85-1.47] | 1.1 [0.86-1.47] | 1.0 [0.84-1.46] | 1.1 [0.87-1.46] | 0.790 |
| GFR CKD-EPI (ml/min) | 56.0 [38-74.4] | 51.0 [32.3-69.5] | 57.5 [38.8-75.62] | 56.0 [38.3-73.9] | 0.660 |
| Hemoglobin (g/dl) | 12.3 [11-13.4] | 12.2 [10.6-13.2] | 12.4 [10.9-13.1] | 12.5 [11.2-13.9] | **0.025** |
| BNP (ng/L) | 261.5 [151.5-406] | - | 256.0 [36-256] | 267.0 [190– 267] | 0.830 |
| NT-proBNP (ng/L) | 1525 [618-2746] | 585.0 [566-585] | 629.0 [368-1574] | 1922.5 [1192-3178] | **< 0.001** |
| Total Bilirubin, mg/dl | 0.82 [0.5-1.35] | 0.98 [0.43-1.26] | 0.89 [0.5-1.5] | 0.75 [0.4-1.15] | 0.830 |
| NYHA class   - Class I - Class II - Class III - Class IV | I 35 (24.5)  II 86 (60.1)  III 19 (13.3)  IV 3 (2.1) | I 10 (27.7)  II 23 (63.9)  III 1 (2.8)  IV 2 (5.5) | I 16 (29.6)  II 33 (61.1)  III 5 (9.3)  IV 0 (0) | I 9 (17.0)  II 30 (56.5)  III 13 (24.5)  IV 1 (1.9) | **0.029** |
| Loop diuretics | 120 (84.5)* | 29 (27.8) | 45 (83.3) | 46 (88.4)* | 0.575 |
| Loop diuretics dosage (mg) | 50.0 [25.0-75.0] | 50.0 [25.0-75.0] | 25.0 [25.0-68.75] | 50.0 [25.0-75.0] | 0.560 |
| Beta-blockers | 75 (69.4)* | 20 (66.7)* | 27 (61.3)* | 28 (82.3)* | 0.127 |
| ACEi/ARB/ARNI | 41 (37.9)* | 9 (30.0)* | 15 (34.1)* | 17 (50.0) * | 0.204 |
| MRA | 29 (26.7)* | 8 (26.7)* | 12 (27.3)* | 9 (26.5)* | 0.997 |
| SGLT2 inhibitors # | 0 | 0 | 0 | 0 | - |
| HFA-PEFF score | 5.5 [4.0-6.0] | 3.0 [4.0-4.0] | 6.0 [5.0-6.0] | 6.0 [6.0-6.0] | **<0.001** |
| TRI-SCORE | 2 [1-3] | 2 [1-3] | 2 [1-3] | 2 [2-4] | 0.234 |
| LVEDD (mm) | 48.0 [44.0-52.0] | 48 [44.0-51.0] | 46.0 [43.0-53.0] | 49.0 [44.5-53.0] | 0.690 |
| LVESD (mm) | 31.0 [27.0-37.0] | 32.0 [28.0-37.0] | 30.0 [26.25-34.0] | 33.0 [26.25-38.0] | 0.290 |
| LVEDV index (ml/m^2^) | 47.0 [40.0-58.5] | 47.0 [39.0-58.0] | 48.0 [40.75-57.04] | 47.0 [38.6-62.0] | 0.990 |
| LVESV index (ml/m^2^) | 19.0 [15.0-25.0] | 20.0 [15.0-25.0] | 18.5 [15.0-23.3] | 19.9 [15.0-26.0] | 0.850 |
| LV Ejection Fraction (%) | 60 [55-63] | 60.0 [53.3-63.0] | 60.0 [56.8-65.0] | 59.0 [54.0-62.0] | 0.210 |
| LVM index (g/ m^2^) | 112.0 [90.0-132.0] | 114.0 [85.0-132.0] | 112.0 [93.0-134.0] | 108.0 [90.0-132.0] | 0.670 |
| MR grade (%)  - None  - Mild  - Moderate  - Moderate to Severe  - Severe | 5 (3.5)  29 (20.2)  43 (30.1)  27 (18.9)  39 (27.3) | 2 (5.5)  7 (19.4)  14 (38.9)  5 (13.9)  8 (22.2) | 1 (0.2)  10 (18.5)  16 (29.6)  11 (20)  16 (29.6) | 2 (3.8)  12 (22.6)  13 (24.5)  11 (20.8)  15 (28.3) | 0.884 |
| Mechanism of MR  - Primary  - Secondary  - Mixed | 40 (42.5)*  35 (37.2)*  19 (20.2)* | 12 (46.1)*  7 (27.0)*  5 (19.2) | 15 (41.7)*  10 (27.8)*  7 (19.4)* | 13 (28.3)*  18 (39)*  7 (15.2)* | 0.613 |
| MR EROA-PISA (cm^2^) | 0.4 [0.26-0.51] | 0.45 [0.36-0.6] | 0.35 [0.22-0.52] | 0.28 [0.14-0.67] | 0.970 |
| MR Vena Contracta (mm) | 5.0 [3.0-6.8] | 5.0 [3.0-8.0] | 4.0 [2.5-7.0] | 5.0 [4.0-6.0] | 0.830 |
| Moderate or Severe aortic stenosis | 13 (9.1) | 0 | 9 (16.7) | 4 (7.5) | **0.023** |
| Aortic regurgitation   - None - Mild - Moderate - Moderate to Severe - Severe | 52 (36.4)  46 (32.2)  32 (22.4)  9 (6.3)  4 (2.8) | 10 (27.8)  12 (33.3)  10 (27.8)  2 (5.5)  2 (5.5) | 21 (38.9)  15 (27.8)  13 (24.1)  3 (5.5)  2 (3.7) | 21 (39.6)  19 (35.8)  9 (17.0)  4 (7.5)  0 | 0.692 |
| Left Atrial Volume Index (ml/m^2^) | 54.0 [42.9-70.5] | 61.0 [41.0-73.0] | 45.5 [38.0-62.0] | 62.0 [49.1-81.0] | **0.001** |
| E/e’ average | 11.0 [9.0-15.0] | 10.0 [6.8-12.0] | 11.0 [9.0-14.3] | 13.0 [10.0-17.1] | **0.020** |
| TR grade  - Severe  - Massive  - Torrential | 128 (89.5)  15 (10.5)  0 (0) | 34 (94.4)  2 (5.6)  0 | 53 (98.0)  1 (2.0)  0 | 41 (77.4)  12 (22.6)  0 | **0.001** |
| TR EROA-PISA (cm^2^) | 0.42 [0.37-0.56] | 0.49 [0.31-0.59] | 0.42 [0.33-0.61] | 0.42 [0.37-0.54] | 0.980 |
| TR Vena Contracta (mm) | 7.8 [6.5-9.0] | 8.0 [6.25-9.75] | 6.0 [6.0-7.0] | 8.0 [7.0-9.0] | **0.005** |
| TR Regurgitant Volume (ml) | 46.0 [36.0-50.0] | 44.0 [31.0-50.0] | 37.0 [32.0-43.0] | 46.0 [36.5-51.5] | 0.252 |
| AP tricuspid annular diameter, mm | 41.0 [39.0-43.0] | 41.0 [39.0-43.5] | 41.0 [39.0-43.5] | 40.0 [38.0-42.5] | 0.572 |
| Indexed AP tricuspid annular diameter, mm/m2 | 23.1 [20.7-25.3] | 22.9 [21.2-25.7] | 23.8 [21.9-25.3] | 22.4 [20.0-25.2] | 0.352 |
| SL tricuspid annular diameter, mm | 40.0 [39.0-43.0] | 42.0 [38.5-44.5] | 40.0 [38.7-42.0] | 40.0 [38.2-42.0] | 0.303 |
| Indexed SL tricuspid annular diameter, mm/m2 | 23.5 [21.2-25.1] | 23.7 [22.1-25.4] | 23.6 [22.1-25.0] | 22.2 [20.6-24.6] | 0.175 |
| Tricuspid tenting height, mm | 2.0 [1.0-4.0] | 2.0 [0.5-4.0] | 2.0 [1.0-3.0] | 3.0 [2.0-4.0] | 0.093 |
| Right Atrial Volume Index (ml/m^2^) | 44.0 [33.0-56.2] | 48.0 [40.0-57.0] | 41.5 [37.8-46.0] | 53.0 [42.5-62.7] | **< 0.001** |
| RV diastolic basal diameter (mm) | 38.0 [35.0-40.0] | 38.0 [35.0-40.0] | 37.0 [35.0-40.0] | 38.0 [36.0-41.5] | 0.056 |
| RV diastolic mid diameter (mm) | 32.0 [28.0-34.0] | 32.5 [29.0-35.0] | 31.0 [28.0-33.2] | 32.0 [27.5-34.0] | 0.360 |
| End Diastolic Area Index (cm^2^/ m^2^) | 9.5 [8.0-10.4] | 9.0 [8.0-10.0] | 9.0 [8.0-11.0] | 9.83 [8.89 -10.5] | 0.350 |
| TAPSE (mm) | 20.0 [18.0-23.0] | 20.0 [19.0-23.0] | 20.0 [19.0-25.0] | 19.0 [17.0-21.7] | **0.010** |
| S’TDI (cm/s) | 12.0 [10.0-13.0] | 12.0 [11.0-13.0] | 12.0 [11.0-14.0] | 11.0 [10.0-13.0] | **0.060** |
| RVFWLS (%) | -24.0 [-27,-20] | -24.5 [-26.7,-20] | -25% [-28,-20.7] | -22% [-25.5,-20] | 0.076 |
| Fractional Area Change (%) | 45.0 [41.0-49.0] | 45.0 [41.0-49.7] | 45.5 [43.7-50.0] | 44.0 [40.0-48.0] | 0.110 |
| sPAP (mmHg) | 40.0 [35.0-45.0] | 36.5 [34.0-45.0] | 37.5 [31.0-44.2] | 40.0 [37.5-45.0] | **0.020** |
| RAP (mmHg) | 5.0 [5.0-10.0] | 5 [5.0-5.0] | 5.0 [5.0-5.0] | 5.0 [5.0-10.0] | **0.014** |
| Values are expressed as number (percentages), mean ± standard deviation or median [interquartile ranges], as appropriate.  * the proportion of patients was calculated on the number of patients with available data.  # most patients were included in the registry before Italian Medicine Agency approved SGLT2 inhibitors for heart failure.  Abbreviations: STR, secondary tricuspid regurgitation; BSA, body surface area; COPD, chronic obstructive pulmonary disease; CAD, coronary artery disease; PCI, percutaneous coronary intervention; CABG, coronary artery bypass graft; TIA, transient ischemic attack; PAD, peripheral arterial disease; HF, heart failure; CRT-D/P, cardiac resynchronization therapy with defibrillator/pacemaker; PM, pacemaker; ICD, implantable cardioverter defibrillator; GFR, glomerular filtration rate; CKD-EPI, Chronic Kidney Disease Epidemiology Collaboration; NT proBNP, n-terminal pro b type natriuretic peptide; NYHA, New York Heart Association; ACEi, angiotensin-converting enzyme inhibitors; ARB, angiotensin 2 receptor blockers; ARNI, angiotensin receptor neprilysin inhibitors; MRA, mineralocorticoid receptor antagonists; SGLT2, sodium-glucose cotransporter-2; HFA-PEFF score, Heart Failure Association-PEFF score; LVEDD, left ventricular end-diastolic diameter; LVESD, left ventricular end-systolic diameter; LVEDV, left ventricular end-diastolic volume; LVESV, left ventricular end-systolic volume; LVM, left ventricular mass; MR, mitral regurgitation; EROA-PISA, Effective Regurgitant Orifice Area-Proximal Isovelocity Surface Area; TR, tricuspid regurgitation; RV, right ventricle; TAPSE, tricuspid annular plane excursion; S’ TDI, Doppler tissue imaging-derived tricuspid lateral annular systolic velocity; RVFWLS, RV free wall longitudinal strain; sPAP, systolic pulmonary arterial pressure, RAP, right atrial pressure. | | | | | |

**Supplemental Table 2. Demographic, clinical, laboratory and echocardiographic characteristics of the five aetiological phenotypes of ventricular secondary tricuspid regurgitation.**

|  | **All VSTR patients**  **(n=505)** | **RV Dysfunction**  **(n=14)** | **HFpEF w/o severe LS-VHD (n=179)** | **HFrEF/HFmrEF w/o severe LS-WHD (n=148)** | **Severe LS-VHD n=144)** | **Precapillary PH**  **(n=20)** | **P value** |
| --- | --- | --- | --- | --- | --- | --- | --- |
| Age (years) | 80 [72-84] | 74.9 [54.8-82.4] | 80.0[74.0-84.5] | 78.8[71.0-84.0] | 80.0[73.0-85.0] | 74.5[71.25-81.8] | 0.156 |
| Male Sex | 252 (49.9) | 9 (64.2) | 70 (50.4) | 90 (60.8) | 77 (53.5) | 6 (30.0) | **< 0.001** |
| Body Surface Area (m^2^) | 1,79 [1.62-1.94] | 1.8 [1.6-2.0] | 1.8 [1.6-2.0] | 1.8 [1.6-2.0] | 1.8 [1.6-1.9] | 1.8 [1.6-2.0] | 0.251 |
| Hypertension | 340 (67) | 9 (64.3) | 123 (68.7) | 105 (70.9) | 94 (65.3) | 9 (45.0) | 0.206 |
| Diabetes | 126 (24.9) | 1 (7.1) | 44 (24.6) | 46 (31.1) | 31 (21.5) | 4 (20.0) | 0.334 |
| Dyslipidemia | 221 (44) | 4 (28.6) | 85 (47.5) | 69 (46.6) | 59 (40.9) | 4 (20.0) | 0.097 |
| Cancer | 36 (7.1) | 1 (7.1) | 13 (7.3) | 15 (10.1) | 4 (2.8) | 3 (15.0) | 0.091 |
| COPD | 90 (17.8) | 1 (7.1) | 31 (17.3) | 29 (19.6) | 20 (13.9) | 9 (45.0) | **0.011** |
| CAD | 139 (27.5) | 6 (42.9) | 41(22.9) | 56 (37.8) | 34 (23.6) | 2 (10.0) | **0.004** |
| Prior PCI | 100 (19.8) | 5 (35.8) | 27 (15.1) | 39 (26.4) | 28 (19.4) | 1 (5.0) | **0.021** |
| Prior CABG | 58 (11.5) | 1 (7.1) | 22 (12.3) | 22 (14.9) | 12 (8.3) | 1 (7.1) | 0.371 |
| Prior surgical valve intervention | 12 (2.4) | 1 (7.1) | 4 (2.2) | 2 (1.3) | 5 (3.5) | 0 | 0.511 |
| Prior transcatheter valve intervention | 6 (1.2) | 0 | 4 (2.2) | 0 | 2 (1.4) | 0 | 0.419 |
| History of Stroke/TIA | 68 (13.5) | 2 (14.3) | 30 (16.8) | 19 (12.8) | 17 (11.8) | 0 | 0.270 |
| PAD | 125 (24.7) | 2 (14.3) | 39 (21.8) | 47 (31.8) | 30 (20.8) | 7 (35.0) | 0.096 |
| History of Atrial Fibrillation | 356 (70.5) | 8 (57.1) | 134 (74.5) | 100 (69.4) | 103 (69.6) | 11 (55.0) | 0.195 |
| Type of Atrial Fibrillation  - Paroxysmal  - Long standing | 88 (24.7)  268 (75.3) | 2 (25.0)  6 (75.0) | 34 (25.4)  100 (74.6) | 16 (16.0)  84 (84.0) | 35 (34.0)  68 (66.0) | 1 (9.1)  10 (90.9) | **0.036** |
| Prior HF Hospitalization | 157 (31.2)* | 4 (28.6) | 52 (29.1) | 50 (33.8) | 48 (33.6)* | 3 (15.0) | 0.446 |
| CRT-P | 13 (2.6) | 0 | 4 (2.2) | 6 (4.0) | 2 (1.4) | 0 | 0.681 |
| CRT-D | 38 (7.5) | 0 | 1 (0.5) | 16 (10.8) | 21 (14.6) | 0 | **<0.001** |
| PM | 94 (18.6) | 4 (28.6) | 30 (16.8) | 29 (19.6) | 29 (20.1) | 2 (10.0) | 0.624 |
| ICD | 36 (7.2) | 0 | 5 (2.8) | 18 (12.2) | 13 (9.1) | 0 | **0.007** |
| Admission creatinine (mg/dl) | 1.3 [0.96-1.85] | 1.2 [0.8-1.6] | 1.1 [0.9-1.7] | 1.5 [1.0-1.9] | 1.5 [1.1-2.1] | 1.4 [0.9-1.7] | **0.001** |
| GFR CKD-EPI (ml/min) | 44.9 [29.3-66.5] | 55.2 [37.0-91.5] | 50.3 [32.0-71.0] | 44.0 [29.0-65.2] | 39.3 [26.9-58.2] | 41.5 [31.0-75.7] | **0.006** |
| Hemoglobin (g/dl) | 11.9 [10.4-13.2] | 13.1 [11.3-14.5] | 12.3 [10.6-13.6] | 12.3 [10.6-13.6] | 11.5 [10.4-12.8] | 11.7 [9.5-13.3] | **0.036** |
| BNP (ng/L) | 431 [238-862] | - | 287 [182-471] | 767 [320-1030] | 641 [306-1250] | - | **0.002** |
| NT-proBNP (ng/L) | 3483 [1663-8558] | 1540 [835-6733] | 2070 [1208-4758] | 5068 [2713-10931] | 4923 [1660-10601] | 7002 [3761-12315] | **<0.001** |
| Total Bilirubin, mg/dl | 0.83 [0.68-1.24] | 0.81 [0.7-0.98] | 0.85 [0.66-1.33] | 0.84 [0.70-1.23] | 0.80 [0.60-1.12] | 1.10 [0.70-1.45] | 0.643 |
| NYHA class  - I  - II  - III  - IV | 92 (18.2)  237 (46.9)  150 (29.7)  26 (5.2) | 4 (28.6)  7 (50)  3 (21.4)  0 | 35 (19.5)  93 (51.9)  47 (26.3)  3 (1.7) | 27 (18.5)*  71 (48.6)*  36 (24.7)*  11 (7.5)* | 15 (10.5)*  59 (41.3)*  56 (39.2)*  11 (7.7)* | 6 (30.0)  5 (25.0)  8 (40.0)  1 (5.0) | **0.031** |
| Loop diuretics | 459 (90.9) | 12 (85.7) | 161(89.9) | 134 (90.5) | 136 (94.4) | 16 (80.0) | 0.220 |
| Loop diuretics dosage (mg) | 50.0 [25.0-100] | 43.8 [25.0-93.8] | 50.0 [25.0-100.0] | 55.0 [30.0-100.0] | 75.0 [50.0-125.0] | 50.0 [50.0-100.0] | **0.005** |
| Beta-blockers | 260 (88.4)* | 6 (66.7)* | 71 (80.0)* | 62 (98.4)* | 110 (91.7)* | 11 (84.6)* | **0.001** |
| ACEi/ARB/ARNI | 167 (57.2)* | 3 (37.5)* | 34 (38.2)* | 52 (82.5)* | 71 (59.2)* | 7 (58.3)* | **<0.001** |
| MRA | 157 (53.8)* | 2 (25.0)* | 31 (34.8)* | 53 (84.1)* | 67 (55.8)* | 5 (41.7)* | **<0.001** |
| SGLT2 inhibitors# | 16 (54.4)* | 0 | 0 | 7 (11.1)* | 9 (7.5)* | 0 | **0.023** |
| HFA-PEFF score | 6.0 [5.0-6.0] | 4.0 [2.5-5.0] | 6.0 [5.0-6.0] | 6.0 [5.2-6.0] | 6.0 [6.0-6.0] | 5.0 [4.0-6.0] | **<0.001** |
| LVEDD (mm) | 52.0 [46.0-58.0] | 48.0 [44.0-52.8] | 48.0 [43.0-53.0] | 56.0 [50.0-60.0] | 55.0 [50.0-61.0] | 45.5 [40.5-51.5] | **<0.001** |
| LVESD (mm) | 38.0 [31.0-45.0] | 33.5 [26.5-35.8] | 33.0 [29.0-39.0] | 44.0 [39.0-50.5] | 39.5 [33.0-46.0] | 28.0 [20.0-31.5] | **<0.001** |
| LVEDV index (ml/m^2^) | 56.7 [43.0-77.0] | 53.0 [35.0-57.9] | 46.0 [38.6-57.0] | 69.9 [53.0-86.5] | 72.0 [49.0-91.3] | 45.9 [34.0-56.9] | **<0.001** |
| LVESV index (ml/m^2^) | 27.0 [19.0-45.0] | 18.0 [15.3-25.5] | 20.0 [15.5-24.0] | 43.0 [30.0-57.0] | 34.0 [21.0-56.3] | 20.0 [12.0-26.3] | **<0.001** |
| LV Ejection Fraction (%) | 50 [37-59] | 56.5 [51.5-59.8] | 57.0 [54.0-61.0] | 36.0 [30.0-43.0] | 50.0 [34.3-60.0] | 55.0 [50.8-62.0] | **<0.001** |
| LVM index (g/ m^2^) | 128 [101-156] | 94 [76-107] | 113 [86-137.] | 136 [117-166] | 141 [117-173] | 109 [90-128] | **<0.001** |
| MR grade (%)  - None  - Mild  - Moderate  - Moderate to Severe  - Severe | 27 (5.3)  131 (25.9)  146 (28.9)  87 (17.2)  114 (22.5) | 1 (7.1)  9 (64.3)  3 (21.4)  1 (7.1)  0 | 15 (8.4)  57 (31.8)  70 (39.1)  37 (20.7)  0 | 9 (6.1)  47 (31.7)  52 (35.1)  40 (27.0)  0 | 2 (1.4)  6 (4.2)  15 (10.4)  7 (4.8)  114 (79.2) | 0  12 (60.0)  6 (30.0)  2 (10.0)  0 | **<0.001** |
| Mechanism of MR  - Primary  - Secondary  - Mixed | 56 (17.9)*  212 (67.7)*  45 (14.4)* | 1 (14.3)*  3 (42.9)*  0* | 19 (17.6)*  54 (50)*  13 (12.0)* | 5 (5.2)*  73 (76.0)*  9 (9.4)* | 30 (22.7)*  75 (56.8)*  23 (17.4)* | 1 (11.1)*  7 (77.8)*  0* | **<0.001** |
| MR EROA-PISA (cm^2^) | 0.32 [0.22-0.4] | - | 0.21 [0.16-0.25] | 0.24 [0.20-0.28] | 0.40 [0.35-0.46] | 0.14 [0.13-0.14] | **<0.001** |
| MR Vena Contracta (mm) | 5.0 [3.0-6.3] | 2.75 [1.5-2.75] | 4.0 [3.0-5.55] | 4.5 [2.5-6.0] | 8.0 [7.0-9.0] | 3.0 [2.48-3.75] | **<0.001** |
| Moderate or severe aortic stenosis | 30 (6.1)* | 0 | 0 | 0 | 30 (20.8) | 0 | **<0.001** |
| Aortic regurgitation   - None - Mild - Moderate - Moderate to Severe - Severe | 198 (40)*  189 (38.2)*  82 (16.7)*  15 (3.0)*  11 (2.2)* | 7 (50)  7 (50)  0  0  0 | 91 (51.7)*  60 (34.1)*  22 (12.5)*  3 (1.7)*  0 | 59 (41.8)*  63 (44.7)*  16 (11.3)*  3 (2.1)*  0 | 33 (22.9)  52 (36.1)  39 (27.1)  9 (6.3)  11 (7.6) | 8 (40.0)  7 (35.0)  5 (25.0)  0  0 | **<0.001** |
| Left Atrial Volume Index (ml/m^2^) | 60.0 [47.0-79.0] | 38.0 [24.5-47.2] | 59.2 [45.0-76.0] | 57.0 [45.6-69.3] | 71.6 [58.5-90.9] | 31.2 [27.9-50.5] | **<0.001** |
| E/e’ average | 14.0 [10.0-19.0] | 9.0 [7.0-12.0] | 13.0 [10.0-18.0] | 15.0 [11.0-19.0] | 15.0 [12.0-20.0] | 10.0 [8.3-11.5] | **<0.001** |
| TR grade  - Severe  - Massive  - Torrential | 418 (82.7)  83 (16.4)  4 (0.8) | 10 (71.4)  3 (21.4)  1 (7.1) | 151 (84.4)  26 (14.5)  2 (1.1) | 122 (82.4)  26 (14.5)  0 | 117 (81.2)  26 (18.1)  1 (0.7) | 18 (90.0)  2 (10.0)  0 | 0.224 |
| TR EROA-PISA (cm^2^) | 0.44 [0.35-0.52] | 0.39 [0.30-0.44] | 0.40 [0.33-0.50] | 0.40 [0.35-0.51] | 0.50 [0.45-0.64] | 0.39 [0.33-0.93] | **0.008** |
| TR Vena Contracta (mm) | 7.5 [6.5-9.0] | 7.0 [6.2-12.8] | 7.0 [6.0-9.0] | 7.0 [6.0-9.0] | 9.0 [7.0-11.0] | 7.0 [6.5-8.5] | **0.001** |
| TR Regurgitant Volume (ml) | 45.0 [35.0-52.0] | 32.0 [31.5-49.0] | 41.0 [35.5-49.0] | 41.0 [33.0-58.0] | 52.0 [42.3-60.0] | 41.0 [35.0-63.5] | 0.143 |
| AP tricuspid annular diameter, mm | 40.0 [38.0-44.0] | 43.0 [39.0-46.0] | 40.0 [38.0-43.0] | 40.0 [37.0-44.0] | 41.0 [39.0-45.0] | 39.0 [38.0-40.5] | 0.058 |
| Indexed AP tricuspid annular diameter, mm/m2 | 23.0 [20.3-25.3] | 23.8 [23.1-25.6] | 22.3 [19.2-24.4] | 21.9 [20.1-24.7] | 23.5 [21.1-26.1] | 23.6 [20.6-25.7] | **0.015** |
| SL tricuspid annular diameter, mm | 40.0 [38.0-44.0] | 41.0 [39.0-45.0] | 40.0 [37.0-44.0] | 40.0 [37.0-44.0] | 42.0 [39.0-45.0] | 40.5 [39.0-42.0] | **0.015** |
| Indexed SL tricuspid annular diameter, mm/m2 | 22.8 [20.6-25.4] | 22.8 [21.7-27.8] | 22.2 [19.6-25.2] | 22.1 [20.1-24.3] | 23.9 [21.5-26.3] | 24.9 [20.6-26.6] | **<0.001** |
| Tricuspid tenting height, mm | 8.0 [6.0-10.0] | 10.0 [7.0-11.5] | 7.0 [6.0-9.0] | 7.0 [6.0-9.0] | 8.0 [6.0-10.0] | 10.0 [6.0-11.0] | **0.015** |
| Right Atrial Volume Index (ml/m^2^) | 51.0 [38.0-69.0] | 49.0 [40.1-96.5] | 47.4 [36.0-65.1] | 50.0 [37.9-65.4] | 53.0 [40.5-72.0] | 64.9 [45.0-87.0] | 0.154 |
| RV diastolic basal diameter (mm) | 42.0 [38.0-47.0] | 46.0 [42.0-51.5] | 42.0 [37.7-46.0] | 43.0 [38.0-48.0] | 42.0 [38.0-47.0] | 47.0 [42.7-50.0] | **0.005** |
| RV diastolic mid diameter (mm) | 35.0 [30.0-40.0] | 39.0 [36.0-46.0] | 35.0 [29.0-39.0] | 33.0 [28.0-39.0] | 36.0 [32.0-41.0] | 39.0 [33.5-47.0] | **0.001** |
| End Diastolic Area Index (cm^2^/ m^2^) | 12.0 [10.0-14.0] | 14.0 [13.0-19.0] | 12.0 [10.0-14.0] | 12.0 [10.0-14.0] | 12.0[10.0-14.0] | 14.5 [12.9-17.9] | **0.001** |
| TAPSE (mm) | 17.0 [15.0-20.0] | 17.5 [13.5-24.0] | 19.0 [16.0-21.0] | 16.0 [14.0-19.0] | 18.0 [16.0-20.0] | 16.0 [13.0-21.0] | **<0.001** |
| S’TDI (cm/s) | 10.0 [8.0-12.0] | 10.0 [8.5-12.0] | 10.0 [9.0-12.0] | 9.0 [7.0-10.0] | 10.0 [9.0-11.0] | 9.0 [7.0-12.0] | **<0.001** |
| RVFWLS (%) | -15.0 [-20,-12] | -16.0[-18.5,-1.4] | -16 [-21.4,-13] | -14.0 [-19,-11] | -15.0[-20.0,12.0] | -15.0 [-20.5,-11.4] | **0.020** |
| Fractional Area Change (%) | 40.0 [35.0-44.0] | 32.0 [24.0-42.0] | 40.0 [37.0-46.0] | 38.0 [33.0-42.0] | 40.0 [35.0-45.0] | 31.0 [26.8-38.8] | **<0.001** |
| sPAP (mmHg) | 50.0 [40.0-60.0] | 35.0 [28.0-58.5] | 47.5 [40.0-56.0] | 46.0 [40.0-56.0] | 55.0 [45.0-63.5] | 68.0 [50.0-80.0] | **<0.001** |
| RAP (mmHg) | 10.0 [5.0 -15.0] | 10.0 [5.0-15.0] | 10.0 [5.0-15.0] | 10.0 [5.0-15.0] | 10.0 [5.0-15.0] | 10.0 [5.0-15.0] | 0.117 |
| Values are expressed as number (percentages), mean ± standard deviation or median [interquartile ranges], as appropriate.  * the proportion of patients was calculated on the number of patients with available data.  # most patients were included in the registry before Italian Medicine Agency approved SGLT2 inhibitors for heart failure.  Abbreviations: STR, secondary tricuspid regurgitation; BSA, body surface area; COPD, chronic obstructive pulmonary disease; CAD, coronary artery disease; PCI, percutaneous coronary intervention; CABG, coronary artery bypass graft; TIA, transient ischemic attack; PAD, peripheral arterial disease; HF, heart failure; CRT-D/P, cardiac resynchronization therapy with defibrillator/pacemaker; PM, pacemaker; ICD, implantable cardioverter defibrillator; GFR, glomerular filtration rate; CKD-EPI, Chronic Kidney Disease Epidemiology Collaboration; NT proBNP, n-terminal pro b type natriuretic peptide; NYHA, New York Heart Association; ; ACEi, angiotensin-converting enzyme inhibitors; ARB, angiotensin 2 receptor blockers; ARNI, angiotensin receptor neprilysin inhibitors; MRA, mineralocorticoid receptor antagonists; SGLT2, sodium-glucose cotransporter-2HFA-PEFF score, Heart Failure Association-PEFF score; LVEDD, left ventricular end-diastolic diameter; LVESD, left ventricular end-systolic diameter; LVEDV, left ventricular end-diastolic volume; LVESV, left ventricular end-systolic volume; LVM, left ventricular mass; MR, mitral regurgitation; EROA-PISA, Effective Regurgitant Orifice Area-Proximal Isovelocity Surface Area; TR, tricuspid regurgitation; RV, right ventricle; TAPSE, tricuspid annular plane excursion; S’ TDI, Doppler tissue imaging-derived tricuspid lateral annular systolic velocity; RVFWLS, RV free wall longitudinal strain; sPAP, systolic pulmonary arterial pressure, RAP, right atrial pressure. | | | | | | | |

**Supplemetal Figure 1.** Kaplan Meier curves for cumulative survival free from the composite outcome according to atrial and ventricular STR in patients managed conservatively during follow-up. Abbreviations: STR, secondary tricuspid regurgitation.


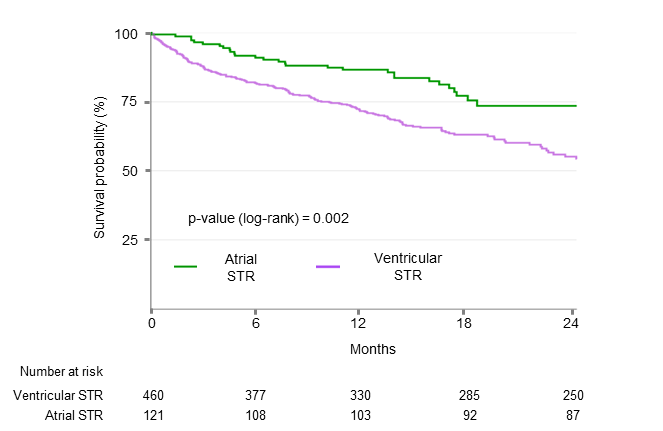


**Supplemental Figure 2.** Kaplan Meier curves for cumulative survival free from the composite outcome according to atrial STR aetiologies in patients managed conservatively during follow-up. Abbreviations: AF, atrial fibrillation, HFpEF, heart failure with preserved ejection fraction.


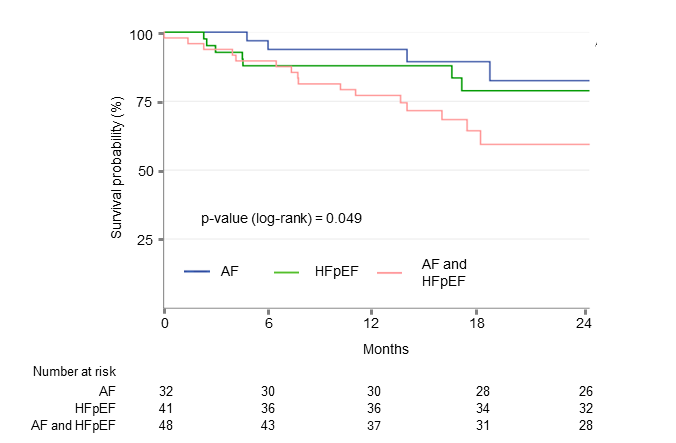


**Supplemental Figure 3.** Kaplan Meier curves for cumulative survival free from the composite outcome according to ventricular STR aetiologies in patients managed conservatively during follow-up. Abbreviations: LS-VHD, left-sided valvular heart disease; HFpEF, heart failure with preserved ejection fraction; RV, right ventricular; HFrEF, heart failure with reduced ejection fraction; HFmrEF, heart failure with mid-range ejection fraction; PH, pulmonary hypertension.


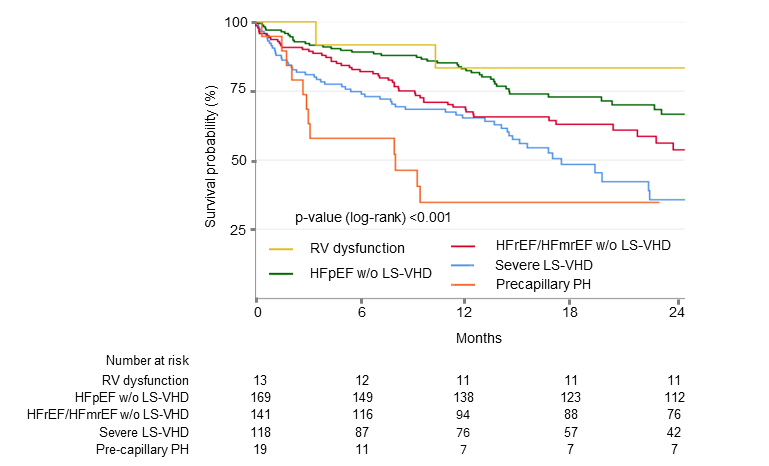

Supplement: Supplementary file 1 — Appendix S1. Supporting Information. [file EJHF-27-1549-s001.docx]
